# Virtual patients in the acquisition of clinical reasoning skills: does presentation mode matter? A quasi-randomized controlled trial

**DOI:** 10.1186/s12909-017-1004-2

**Published:** 2017-09-15

**Authors:** Fabian Schubach, Matthias Goos, Götz Fabry, Werner Vach, Martin Boeker

**Affiliations:** 1 Institute for Medical Biometry and Statistics, Faculty of Medicine and Medical Center - University of Freiburg, Stefan-Meier-Str. 26, 79104 Freiburg i. Br., Germany; 2 Department of General and Visceral Surgery, Helios Klinik Müllheim, Heliosweg, 79379 Müllheim, Germany; 3 Department of Medical Psychology and Medical Sociology, Faculty of Medicine and Medical Center - University of Freiburg, Rheinstr. 12, 79104 Freiburg i. Br., Germany

**Keywords:** Virtual patients, Clinical reasoning, Instructional methods, Key features, Undergraduate medical education

## Abstract

**Background:**

The objective of this study is to compare two different instructional methods in the curricular use of computerized virtual patients in undergraduate medical education. We aim to investigate whether using many short and focused cases – the key feature principle – is more effective for the learning of clinical reasoning skills than using few long and systematic cases.

**Methods:**

We conducted a quasi-randomized, non-blinded, controlled parallel-group intervention trial in a large medical school in Southwestern Germany. During two seminar sessions, fourth- and fifth-year medical students (*n* = 56) worked on the differential diagnosis of the acute abdomen. The educational tool – virtual patients – was the same, but the instructional method differed: In one trial arm, students worked on multiple short cases, with the instruction being focused only on important elements (“key feature arm”, *n* = 30). In the other trial arm, students worked on few long cases, with the instruction being comprehensive and systematic (“systematic arm”, *n* = 26). The overall training time was the same in both arms. The students’ clinical reasoning capacity was measured by a specifically developed instrument, a script concordance test. Their motivation and the perceived effectiveness of the instruction were assessed using a structured evaluation questionnaire.

**Results:**

Upon completion of the script concordance test with a reference score of 80 points and a standard deviation of 5 for experts, students in the key feature arm attained a mean of 57.4 points (95% confidence interval: 50.9–63.9), and in the systematic arm, 62.7 points (57.2–68.2), with Cohen’s d at 0.337. The difference is statistically non-significant (*p* = 0.214). In the evaluation survey, students in the key feature arm indicated that they experienced more time pressure and perceived the material as more difficult.

**Conclusions:**

In this study powered for a medium effect, we could not provide empirical evidence for the hypothesis that a key feature-based instruction on multiple short cases is superior to a systematic instruction on few long cases in the curricular implementation of virtual patients. The results of the evaluation survey suggest that learners should be given enough time to work through case examples, and that caution should be taken to prevent cognitive overload.

**Electronic supplementary material:**

The online version of this article (10.1186/s12909-017-1004-2) contains supplementary material, which is available to authorized users.

## Background

Assigning medical diagnoses to the complaints, problems and symptoms reported by patients – a process commonly referred to as “clinical” or “diagnostic reasoning” – is at the core of what physicians do in their daily routine [1–3]. The processes inherent in diagnostic expertise and its acquisition have been studied for more than 40 years now. When dealing with routine cases, clinical experts tend to make extensive use of sub-conscious, non-analytical reasoning strategies that essentially depend on matching newly encountered patient cases to previously seen ones (“System 1”, pattern recognition, intuitive reasoning, script instantiation, and others), and to gather only a reduced set of clinical information in order to establish a diagnosis. By contrast, deliberate, analytical reasoning (“System 2”) in experts is mostly restricted to non-routine cases, e.g. rare diseases and exceptional case manifestations [3–6]. Dual process theory describes in more detail how the two types of processes interact [3, 4].

Another crucial finding from empirical research has been termed the “context specificity” of clinical reasoning which refers to the observation that there is no significant transfer of diagnostic expertise from one clinical domain to another. Thus, generic “problem-solving skills” do not exist. Rather, competence in solving clinical problems is highly dependent on the content of the problem [5–9]. Furthermore, given the inertness of knowledge and the many factors that impede transfer to clinical scenarios [9, 10], successful clinical reasoning does not develop by accumulation of factual knowledge, but by working through a large number of clinical cases, thus enabling learners to compare and contrast the variable appearance of different disease entities.

Two important consequences for medical education emerge: First, any effective strategy to foster clinical reasoning capacities in medical students must rely on teaching around multiple and varied examples of clinical cases [4–11]. Second, a reliable and valid assessment of clinical reasoning skills requires sufficient sampling of cases across different domains. To achieve this in a reasonable amount of time, it is necessary to focus on the crucial elements of a problem or case, its “key features” [12–14]. This “key feature (KF) principle” – i.e., the principle of focusing on important elements of multiple short cases in order to allow for sufficient sampling, rather than working up a single or very few lengthy cases completely – has originally been developed for assessment, although it is conceivable to apply it to teaching as well.

Based on these considerations, Cook and Triola [15] have presented convincing arguments that virtual patients (VPs), i.e., electronic (computer) simulations of patient cases, are especially well suited to promote clinical reasoning skills in students. As opposed to real patients, VPs can be used in a structured and standardized manner by selecting the sequence and features of cases that are considered optimal for educational purposes. Yet, little is known about how to best incorporate VPs into the curriculum, and only few attempts have been made so far to investigate effective and efficient implementation strategies [15–17]. More generally speaking, there is still a considerable lack of empirical research on how to teach clinical reasoning [18].

As outlined above, the KF principle has been well established for use in assessment [12–14]. In teaching, however, case-based instruction often relies on working up few cases completely and systematically. Students are required to obtain a complete history and physical exam as well as appropriate additional clinical information such as laboratory results and imaging studies, before proceeding to formulate diagnostic hypotheses [4, 19]. Yet, as Bordage pointed out, “thoroughness is not a good predictor of diagnostic accuracy”. Clinical teachers should rather encourage their students to concentrate on “smaller, more focused, and discriminating set of symptoms and signs” when approaching real or simulated patient cases [20]. Bordage recommends, in other words, that focusing on important elements of multiple short cases, i.e. KF-based instruction, should be preferred over the more traditional systematic and comprehensive instruction on few long cases.

There are several reasons to assume that this suggestion is well-founded. First, a focused approach simply allows for working through more cases in the same time which, as mentioned above, is essential for acquiring diagnostic expertise. Second, it mimics the expert’s working method more closely than comprehensive “from A to Z” data gathering [5–7]. Finally, it addresses the problem that learners have difficulties discriminating relevant from irrelevant aspects of a case and are easily distracted by secondary information such as the occupation or appearance of a patient which is typically of lesser importance. Generally, knowledge tends to interact with the context in which it has been acquired [21, 22]. Thus, successful educational strategies aim to overcome this context-dependency and to make the knowledge more flexible by decontextualizing it in order to foster transfer. KF-based instruction has the potential to target this issue [8, 23].

The objective of this work is to investigate whether working on many short and focused cases – the KF principle – is more effective for the learning of clinical reasoning skills than working on few long and systematic cases.

## Methods

### Trial design

We conducted a controlled, non-blinded parallel-group intervention study at the Faculty of Medicine and Medical Center of the University of Freiburg, Freiburg, Germany, with undergraduate medical students working on the differential diagnosis of acute abdomen. Students were allocated to one of the two trial arms at a ratio of 1:1 on the basis of pre-determined, quasi-random learning group numbers, thus resulting in a quasi-randomization. Details of the allocation method are given below.

The educational tool used – VPs – was the same in both arms, but the instructional method differed: In one arm, students worked through four cases per session in 13 min per case, with the instruction following the KF principle, i.e., orienting the students towards focusing only on important similarities and differences (“KF arm”). In the other arm, students worked through one long case (50 min.) per session, with the instruction orienting the students towards working in a comprehensive and systematic manner (“systematic arm”).

The effectiveness of the training was measured with a script concordance test (SCT), a case-based instrument designed to assess the data interpretation component of clinical reasoning skills. Our hypothesis was that students in the KF arm would achieve better results in this test than students in the systematic arm.

### Participants and setting

Eligible participants were students in their fourth and fifth year of medical school who completed the surgical clerkship within a six-week period in June/July 2010. All the students who passed the surgical clerkship in this period were included in the study. The surgical clerkship is a mandatory part of the clinical training of medical students in Germany as provided by the German Medical Licensure Act. In Freiburg, it is scheduled in the fourth or fifth year of medical school. The clerkship comprises several courses, surgical skills training, supervised patient encounters and seminars that the students complete in rotation. The seminars cover different topics in General and Visceral Surgery, and are based on different teaching methods that are largely at the discretion of the individual teacher, predominantly lectures or educational discussions.

The intervention was conducted at the occasion of two of these seminars. A blended-learning curriculum based on VPs covering the topic of acute abdomen was designed for the two 90-min sessions following established guidelines for curriculum development [24]. During these sessions, students were allocated to one of the two instructional methods – KF-based vs. systematic – as explained below. Students of each instructional method, i.e., of each trial arm, were taught in separate seminar group sessions. A third session on the topic of gastrointestinal bleeding was based on patient demonstration and educational discussion without VPs and, within the framework of our study, served to test for homogeneity of the trial arms, i.e., for equality of performance in a subject area other than that of the interventions. In this third session, students of both arms were taught together in a joint session. Moreover, the measurement (SCT) and the evaluation survey were completed on this occasion. In order to obtain the required number of study participants, the study was conducted in three waves, each involving two groups of students in the KF arm and two groups of students in the systematic arm.

**Fig. 1:**
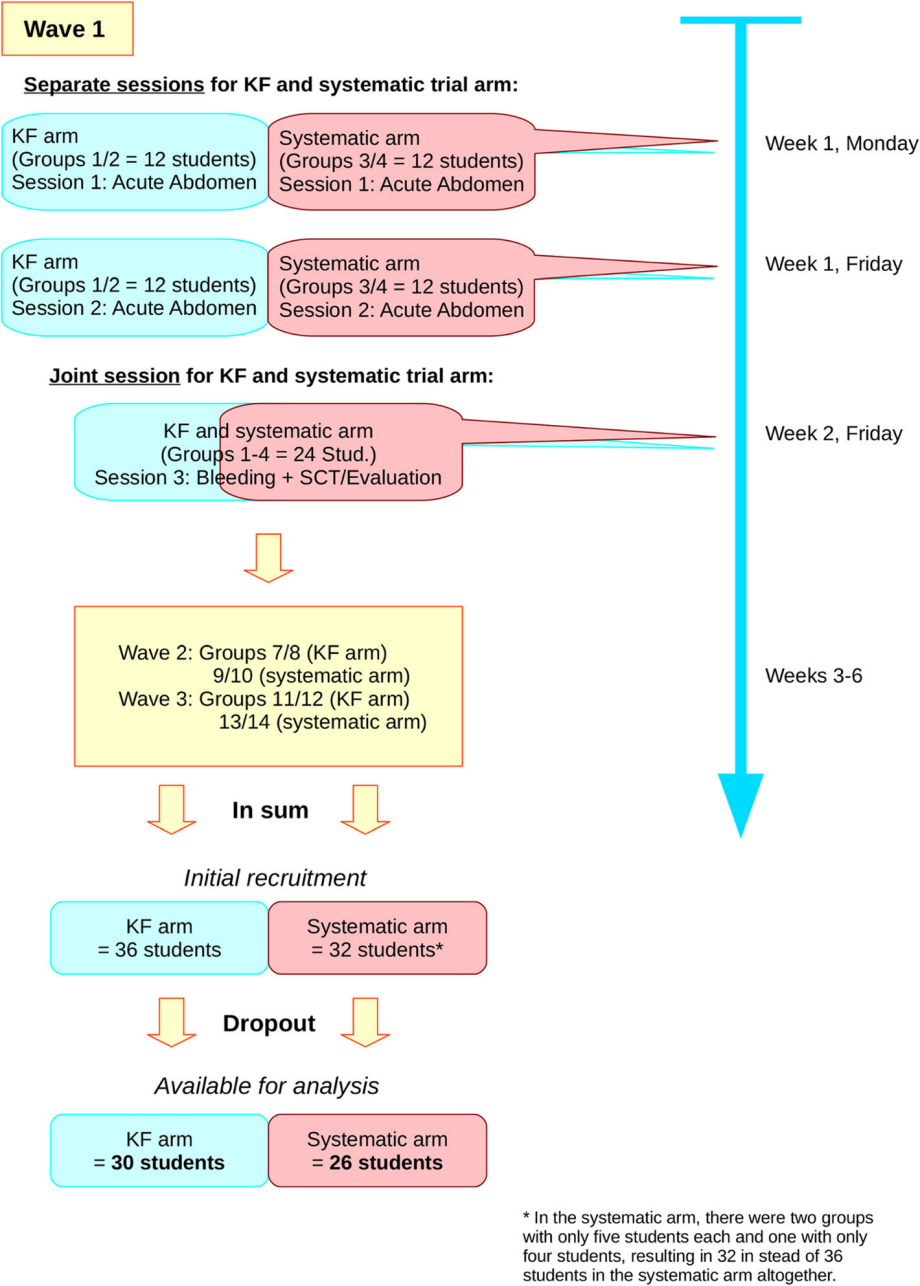
Study design and realization: Timeline and methodology

A visualization of this is provided in Fig. 1, along with further details which we will elaborate on below.

### Determination of sample size

To determine the required sample size for detecting a medium effect (Cohen’s d = 0.75), we conducted a power calculation for a two-sided two-sample t-test, with a significance level set at 0.05, and power at 0.8. The calculation revealed that we would need about 29 students in each trial arm.

### Allocation method

Allocation of students to either the KF or the systematic trial arm was based on the grouping that had been prearranged by the students’ office of the Department of General and Visceral Surgery for the organization of the surgical clerkship as a whole. Following a list in alphabetical order, the office had assigned students to small groups of six, where the first six students in the list were assigned to group 1, the next six to group 2, etc. The first wave of our experiments included group 1–4, the second included group 7–10 and the third included group 11–14. Each wave consisted of the above-mentioned three seminars, i.e., two sessions on acute abdomen and one on gastrointestinal bleeding, with the latter session including the measurement. We defined in advance that the low group numbers of each wave (i.e., 1/2, 7/8, and 11/12) would constitute the KF arm, and the high group numbers (i.e., 3/4, 9/10, and 13/14) would constitute the systematic arm (Fig. 1). This allocation decision was made prior to seeing any students’ names, and thus without any knowledge about who belonged to which learning group or who might be better suited for this or another learning method.

Nevertheless, we are aware that this method of allocating students is not a technically exact randomization as specified, for instance, in the CONSORT statement [25]. Rather, it corresponds to the definition of quasi-randomization as found in the Cochrane Handbook for Systematic Reviews, Box 13.4.a [26]. The fact that the allocation depended, at least to a certain extent, on alphabetical order and on pre-specified blocks, and that the students, therefore, completed other courses in a similar line-up, theoretically results in a potential to influence the results by trial arm. In order to account for this, we applied a random-effects model for adjustment of the *p* values in addition to the regular analyses of the trial arms. Furthermore, we followed two approaches to demonstrate a priori homogeneity of academic performance in both arms: first, as outlined above, by assessing clinical reasoning skills (via SCT performance) in a control topic (gastrointestinal hemorrhage) where all students had been taught together in a joint session without VPs. Second, we looked at the participants’ results in a multiple-choice test on factual knowledge in visceral surgery at the end of the semester.

### Intervention

In the two sessions covering the acute abdomen, students of both arms worked in a blended-learning curriculum based on the Think-Pair-Share principle outlined by Lyman [27], incorporating VP cases from a web-based e-learning tool, the INMEDEA simulator [28]. The INMEDEA simulator is an interactive simulation where the user, by means of a standard web browser, can navigate through a clinic environment and act as “virtual physician”. In different departments, the user can see VPs, take the patient’s history by asking questions from a database, perform a detailed physical examination, order from a wide range of laboratory tests and imaging studies, and finally suggest a diagnosis and a treatment. More details including a demo version can be found online [28]. For our study, a PC lab was equipped with access to the simulator, and students were asked to work through VP cases individually prior to entering a group discussion moderated by a senior clinical teacher. The group discussion served to compare and contrast structural similarities, differences and essential elements of the cases. The actual intervention took place during the individual working phase of the two “acute abdomen” sessions where each student had access to the e-learning platform via his or her own computer. It was in this phase that the instruction differed between the trial arms.

In each of the two seminar sessions, students in the KF arm were given the above-mentioned four VP cases (see Table 1) and were told to work through all of them. Additionally, we gave each student a working sheet in order to provide clear and standardized instructions. On this sheet, they were explicitly instructed to get a general idea and overview of each patient case, and to focus on its key features, rather than to work through all aspects of the case systematically. Specifically, via the sheet, they were prompted to focus on the following selective elements: key characteristics of the chief complaint in the patient’s history such as the progression of symptoms over time, findings on clinical examination of the abdomen, findings on abdominal ultrasound, and signs of inflammation like fever, elevated white blood cell count and C-reactive protein; if appropriate, signs of gastrointestinal blood loss, signs of cholestasis and findings on abdominal CT scan. After that, students were asked to formulate a working diagnosis and, if appropriate, differential diagnoses. The wording of the instructions was explicitly directing the students toward gaining a brief overall impression of each case, focusing on essential elements, and avoiding extensive data gathering. They were requested to spend a maximum of 13 min on each single case and, after this time had elapsed, to turn toward the next case even if they had not fully completed the previous one.

**Table 1:** VP cases covered in the two seminar sessions on the acute abdomen

Session	VP cases
Session 1: Inflammatory diseases	Appendicitis
	Diverticulitis
	Cholecystitis
	Crohn’s disease
Session 2: Non-inflammatory diseases	Ileus due to mesenteric ischemia
	Ruptured abdominal aortic aneurysm
	Ectopic pregnancy
	Ureteral colic

Students in the systematic arm were randomly assigned to only one of the four VP cases. They were also given a working sheet but were not informed in advance about any key features, i.e. they were not directed toward the above-mentioned specific signs and symptoms. Instead, they were given 50 min of time and were explicitly instructed to proceed thoroughly, systematically, and step by step: to take the patient’s history, perform a physical examination, come to a diagnostic hypothesis, order diagnostic tests targeted at confirming the working diagnosis or ruling out competing hypotheses, and finally suggest a treatment. If necessary, they were asked to look for additional information on their patient’s illness via electronic resources (E-Books, databases). Here, the wording of the instructions had an explicit focus on a systematic procedure and on comprehensive data gathering. Students in the systematic arm were also given individual access to the platform after the seminar so that they could work through the VP cases they had not seen themselves.

The group discussion phase was identical in both trial arms. Here, within about 10 minutes, small groups of two to three students, respectively, were assigned to one VP case (in the systematic arm, to the case the students had worked on) and were prompted to bring together essential findings in the history and examination of “their” (virtual) patient. After that, all 12 students entered a discussion in plenary moderated by the clinical teacher. Following the principle of “Compare and Contrast”, this discussion served to identify important structural similarities and differences of all four VP cases and, thereby, enhance the students’ understanding of the causes and mechanisms underlying an acute or unclear abdomen.

### Description of the similarity of interventions

The followings steps were taken to ensure a maximum of similarity of the interventions except for the intended differences:

- We recorded a demonstration video in order to give all the students identical instructions on how to use the simulator. The video was shown to students prior to the first seminar session.
- We maintained identical time intervals between the first, the second and the third session, i.e., between the interventions and the test, respectively.
- All the sessions were held by the same clinical teacher.
- Access to the VP cases used in the interventions was limited to our teaching sessions, that is, these VPs were not accessible via the standard university license so that none of the students would be familiar with any of the cases in advance. Access rights to individual VPs were configured so that each student was only able to see those VP cases he or she was assigned to.

### Outcomes

The primary outcome was the students’ clinical reasoning skills regarding the topic of acute abdomen as measured by a script concordance test, an instrument for the assessment of clinical data interpretation, an aspect considered crucial in the clinical reasoning process [29, 30]. The test had previously been developed for the specific purpose of assessing clinical reasoning in the context of acute abdomen. Established guidelines [2, 31, 32] were carefully followed in the development and validation of the instrument.

The SCT is a case-based test consisting of multiple short scenarios where the examinee has to interpret new pieces of information against the background of a proposed working diagnosis or treatment option using a five-point Likert scale. An example of this is given in Table 2.

**Table 2:** Example of an SCT case with two questions

A 25-year-old, apparently sick female patient is presented to you in the emergency room by her boyfriend. She is complaining of nausea and severe pain in the right lower abdomen		
If you were thinking of the following diagnosis...	...and then find...	...then your hypothesis becomes...
Acute appendicitis	Patient vomits in the emergency room	-2-1 0 +1 +2
Ectopic pregnancy	Acute, sudden onset of pain two hours ago	-2-1 0 +1 +2

-2=ruled out or almost ruled out

-1=less probable

0=neither more nor less probable

+1=more probable

+2=certain or almost certain

The standard scoring method for the SCT is called the aggregate method (AGG) where credit is awarded not on an absolute basis, but in relation to a panel of reference experts in the respective domain, in our case, visceral surgery. Full credit is awarded for the modal answer of each question – i.e., the response item that the majority of the experts chose –, but also partial credit is given for answers chosen by other experts – depending on their proportion. We have used AGG because it is the most widely used method [2, 29, 31]. However, the optimal scoring procedure for the SCT is still under discussion [29, 33–36]. We have focused on this debate in some more detail elsewhere [37].

After scoring of the single items, a raw summary score was obtained by summing up over all cases. Items with negative item-total correlation were then removed until item-total correlations were positive. As recommended by Charlin et al. [38], the item-based score was averaged to obtain case-based scores. Cases with negative “case-total correlations” were removed until all were positive.

In order to make the scores interpretable and comparable, the raw summary score was normalized such that the mean performance of the experts is set at 80 points, and the standard deviation at 5 points. These standard values are arbitrary but allow for comparability of the abstract results of SCTs with conventional performance tests. This normalization is a Z transformation and has been suggested by Charlin et al. [38].

In addition to their clinical reasoning skills, we assessed the students’ motivation and perceived efficacy with regard to the new blended learning method by an established instrument [39] (based on the Illinois Course Evaluation Questionnaire [40]) that was adapted for computer-based learning in medicine. In this questionnaire, the students were given positive as well as negative (critical) judgments of the seminar, and were asked to indicate their degree of accordance with these statements using a five-point Likert scale ranging from 1 = “fully agree” to 5 = “fully disagree”. The instrument has five dimensions: relevance and usefulness of the material; teacher’s behaviour towards the students; adequacy of difficulty and quantity of the material; methodology, structure and clarity of the seminar; involvement/commitment of the students. Each dimension contains five items (statements).

The SCT and the questionnaire were administered right after the third seminar session on gastrointestinal bleedings, corresponding to 11 days after the first and 7 days after the second session on the acute abdomen. The scheduled date of the SCT was not announced to the students beforehand. However, at the outset of the test, they were informed and guaranteed that the results were analyzed in pseudonymized form and with their informed consent only, and that the test results would have no consequences whatsoever for their grades or for passing the seminar or the surgical clerkship. For administrative reasons, SCT results could not be communicated to the students individually. Hence, as its essential function, the SCT was part of our research project. For our study participants, it was neither summative (since it did not affect their academic record) nor explicitly formative in nature (since they did not get feedback on its results).

### Statistical methods

Statistical analyses were done using R version 3.2.2 [41] and Stata version 13.1 [42]. In order to address the primary research question whether there is a significant difference in SCT performance of the KF and the systematic arm, the following calculation steps were carried out:

- Descriptive statistics, including line plots with bootstrap confidence interval as well as conventional boxplots
- Verification of the normal distribution assumption
- Two-sided two-sample t-test, with the significance level set at .05, aiming at testing the null hypothesis that the students’ mean score in the KF trial arm equals that in the systematic trial arm

Two different variants of analysis were carried out, namely, Intention-to-treat (ITT) and Per-Protocol (PP) analysis [25].

With regard to the evaluation questionnaire, the scale for positive items was first inverted, so that high scores correspond to a positive judgment, and low scores to a negative (critical) judgment of the item. An average score for each dimension was then calculated for both trial arms. Finally, a t-test based on ITT was applied to all five dimensions.

## Results

### Script concordance test

In total, our SCT contained 24 cases with 66 items nested within the cases: 18 cases (52 single questions) on acute abdomen and 6 cases (14 single questions) on gastrointestinal hemorrhage. According to the described criteria, 7 single questions had to be deleted from the total test. No case had to be deleted, thus leaving 24 cases with 59 single questions in the total test; 18 cases/47 single questions on acute abdomen and 6 cases/12 single questions on gastrointestinal hemorrhage. Cronbach’s alpha was 0.78. Experts (80 points, 95% CI = [77.1;82.9]) significantly outperformed the combined student group (59.5 points, 95% CI = [50.7;68.2]) by four expert standard deviations (*p* < 0.001). SCT performance was not correlated to MCQ performance (*r* = 0.11).

### Participant recruitment

**Fig. 2:**
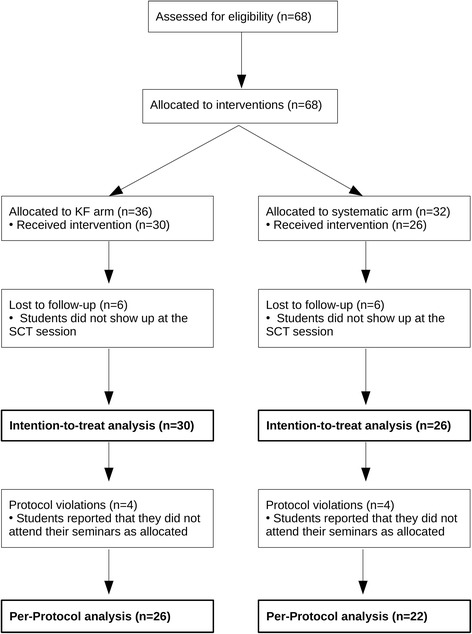
CONSORT diagram of the study

The participant flow is depicted in Fig. 2.

After recruitment and allocation as described in the Methods section, 68 students were included, 36 in the KF arm and 32 in the systematic arm.

We had six cases of missing data in each trial arm because these students did not take part in the SCT and evaluation survey, i.e., in the third seminar session. We do not know whether these students missed only the SCT session or other seminar sessions as well, since central registers to record attendance are not taken in Freiburg – students only collect individual route cards. Hence, we were not able to track students individually after subscription to the surgical clerkship, and thus after allocation to the trial arms. As a consequence, 30 students in the KF arm and 26 students in the systematic arm were available for what we call intention-to-treat (ITT) analysis.

Of these, four students in each arm did not adhere to the protocol. As checked by the authors, these students had either missed one or both seminars on the acute abdomen, or they had attended one seminar session in the KF and one in the systematic arm. All other students attended their seminars as allocated. As a consequence, 26 students in the KF arm and 22 in the systematic arm were available for what we call Per-Protocol (PP) analysis.

### Baseline data

Students were generally between 22 and 30 years old. Roughly two-thirds of our study participants were female, and one third male, corresponding to the general population of medical students in Freiburg at the time of our investigation.

Students of both arms did not differ in their performance in an MCQ test on factual knowledge in visceral surgery at the end of the semester. Out of a maximum of 40 points, students in the KF arm achieved a mean score of 33.71 (95% CI = [32.19;35.22], SD 3.06), and in the systematic arm, 33.26 (95% CI = [31.83;34.69], SD 3.34), thus differing by 0.44 points (95% CI = [−1.73;2.61], Cohen’s d = 0.138, *p* = 0.681).

**Fig. 3:**
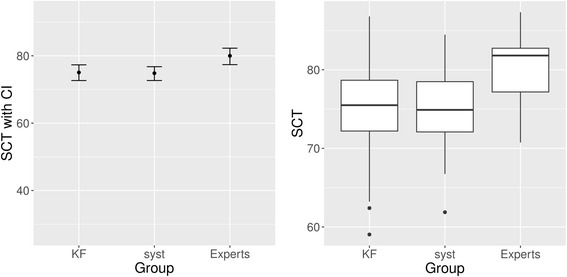
SCT results. Subscale gastrointestinal hemorrhage. KF, key feature arm; syst, systematic arm. Left side: Simple line plot, indicating point estimate with 95% confidence interval. Right side: Boxplots, indicating minimum score, 1st quartile, median, 3rd quartile, maximum score, and potential outliers

Similarly, as shown in Table 3 and Fig. 3, students of both arms achieved almost identical results in the SCT subscale on gastrointestinal hemorrhage, the control topic where all students had been taught together without VPs.

**Table 3:** SCT results. Subscale gastrointestinal hemorrhage

	Mean [95% CI]	SD	
Experts (*n* = 16)	80 [77.1;82.9]	5.0	
KF arm (n = 30)	75.1 [72.6;77.5]	6.5	
Systematic arm (n = 26)	74.8 [72.7;77.0]	5.4	
	Difference [95% CI]	Cohen’s d	p
KF vs. systematic arm: Intention-to-treat	0.23 [−3.01;3.46]	0.038	0.888
KF vs. systematic arm: Per-Protocol	0 [−3.48;3.48]	0.000	1

### SCT results “acute abdomen”

**Fig. 4:**
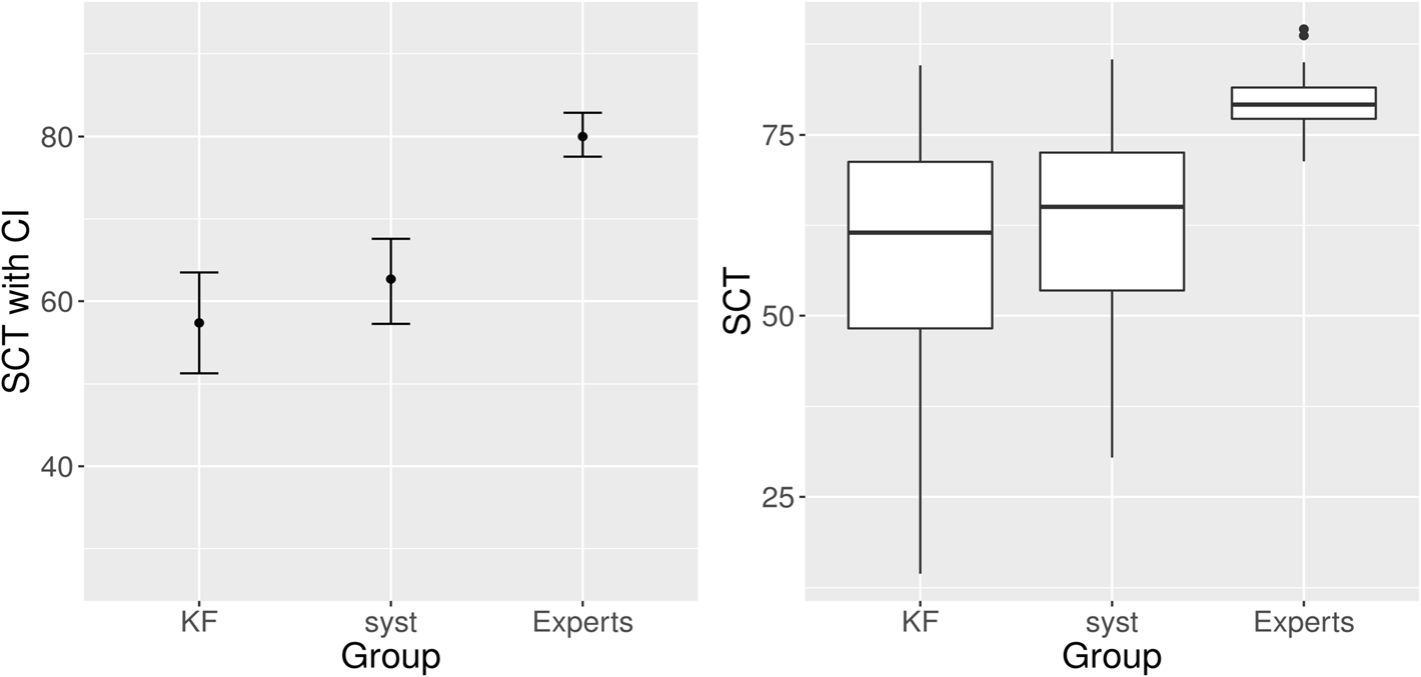
SCT results. Subscale acute abdomen. KF, key feature arm; syst, systematic arm. Left side: Simple line plot, indicating point estimate with 95% confidence interval. Right side: Boxplots, indicating minimum score, 1st quartile, median, 3rd quartile, maximum score, and potential outliers

In the SCT subscale on the intervention topic of acute abdomen, there is a slight numerical, but non-significant advantage for the systematic arm. The exact results are shown in Table 4 and Fig. 4.

**Table 4:** SCT results. Subscale acute abdomen

	Mean [95% CI]	SD	
Experts (n = 16)	80 [77.1;82.9]	5.0	
KF arm (n = 30)	57.4 [50.9;63.9]	17.4	
Systematic arm (n = 26)	62.7 [57.2;68.2]	13.7	
	Difference [95% CI]	Cohen’s d	p
KF vs. systematic arm: Intention-to-treat	−5.32 [−13.79;3.15]	−0.337	0.214
KF vs. systematic arm: Per-Protocol	−5.47 [−14.81;3.88]	−0.335	0.245

Application of the random-effects model to the analyses further shows that the resulting *p* values virtually do not change, suggesting that our method of allocating the students in groups has not influenced the results.

### Evaluation survey

**Fig. 5:**
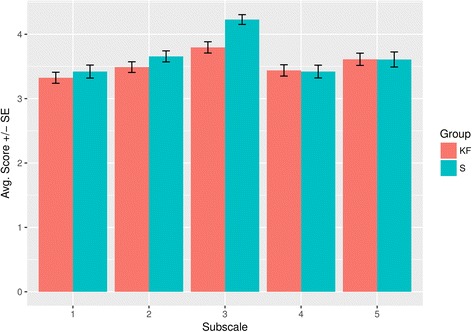
Results of the evaluation survey. Dimensions of the questionnaire: 1 = Relevance and usefulness of the material; 2 = Teacher’s behaviour towards the students; 3 = Adequacy of difficulty and quantity of the material; 4 = Methodology, structure and clarity of the seminar; 5 = Involvement/Commitment of the students

In the evaluation questionnaire, students in the KF arm tended significantly more toward a negative overall judgment on the subscale dealing with the difficulty and quantity of the material (*p* < 0.001). An overview of the results is provided in Fig. 5.

## Discussion

The aim of this study was to investigate whether a focused, KF-based instruction on multiple short VP cases is more effective for the learning of clinical reasoning skills than systematic and comprehensive instruction on few long VP cases. The primary outcome – clinical reasoning – was measured by an SCT. The main result is that there is no statistically significant difference between the two instructions. In contrast to what we expected, a non-significant tendency might even indicate an advantage for the systematic arm.

First, it must be acknowledged that clinical reasoning skills are a multifaceted construct that includes a multitude of competencies including sub-processes such as data gathering, data processing, data interpretation, and decision making. The SCT only accounts for the measurement of the data interpretation component. In this regard, it is a simplification to equate clinical reasoning skills with SCT scores. However, SCT results are at least a good approximation to estimate students’ clinical reasoning abilities.

### Limitations

A potentially confounding issue of our study is related to its design. As stated in the methods section, students in the systematic arm had the possibility to attend additional training after the exposure to the interventional training session by working through the cases they had not seen themselves. We had to introduce this option for the students in the systematic arm to comply with ethical considerations and it was not possible to postpone this additional training to a point in time after the measurement. However, by recording who attended the additional training we documented this source of confounding: 8 students participated in additional training, 14 students did not participate, and for 4 students participation is unknown. Regarding the students for whom participation status is known, we observed a non-significant advantage for the students who attended additional training, compared to those who did not. The trend for students with extra training to expose better SCT scores probably is attributable only in part to the training. Rather, it might largely reflect the selection of students who deliberately attended extra training. The main argument for the validity of our conclusion is that even the subgroup of students in the systematic arm who did not attend the extra training has better SCT results than the students in the KF arm. Admittedly, this argument relies on post-hoc analyses that do not have sufficient power. However, with regard to the 8 students with extra training – presumably high-performing students in the first place – it appears unlikely that, if they had not attended extra training, they would have scored so low as to produce a significant result in favour of the KF arm. But ultimately, we cannot exclude that a different result might have emerged without this limitation, and thus the following elaborations must be viewed against this background.

Another limitation of our study is that the clinical teacher who held all the seminar sessions was involved in designing the study and, hence, was not blinded to the research question and hypothesis. Since our study design implied a significant amount of additional teaching time – due to splitting the seminar groups in two halves – we were not able to recruit a blinded teacher in this clinical environment already characterized by exceedingly high work-load. As for the students, they were probably blind in the first wave of our experiments since we did not give them detailed information about the purpose and the structure of the interventions. However, it is possible that students in the later waves of our experiments have learned something about it from their fellow students in the earlier waves. Thus, we cannot reliably regard the study as blinded.

Furthermore, students of both trial arms achieved substantially better results on the control topic of gastrointestinal bleeding as compared to acute abdomen, due to the SCT being easier on the former subscale. This slightly weakens our evidence for the students’ being a priori equal in their clinical reasoning performance. The interpretation of the MCQ as a baseline measure is limited by the fact that it was administered after the intervention and may include few items on its topic (acute abdomen). However, considering that it also covers a wide range of other topics from the entire domain of visceral surgery, this effect is probably very minor.

Another limitation arose from dropouts. As outlined above, out of the 68 students who initially subscribed to the surgical clerkship and were thus allocated to the trial arms, there were six in each arm who did not take part in the SCT. For the above-mentioned organizational reasons, we do not know exactly what happened to these students between allocation and test, i.e., if and how many interventions they missed, and for what reason. However, what we do know from similar clerkships is that students sometimes simply do not attend the courses they subscribed to, for private, health or other reasons. Furthermore, in order to get their certificate for the clerkship, students have to attend at least 85% of the seminars, and we also know from experience that some students do not show up anymore once they have fulfilled this 85% minimum requirement. We strongly believe that reasons like these account for the six missing students in each trial arm, which makes a systematic bias very unlikely.

### Interpretation

Having set the background of potentially limiting factors, a possible explanation for the observed lack of difference between the arms concerns the relatively short duration of our investigation. According to script theory and other important models, the development of clinical reasoning abilities is rather a long-term process. Thus, it is quite possible that the number and duration of case exposure has a relevant effect on student learning, but that it is simply not detectable after two to three seminars. In order to yield a relevant benefit, covering more case examples over longer time periods might be required.

Furthermore, we have some hints suggesting that the level of difficulty and complexity was very high, particularly for the students in the KF arm. There is, first, the fact that both the content – acute abdomen – and the teaching method – VPs – were entirely new to the students. Second, five students in the KF arm versus only one in the systematic arm – in other words, five times as many KF arm students – stated in the evaluation that the way they were expected to work through the VP cases was too fast for them. Third, while not capturing this systematically in terms of an outcome parameter, we observed that during the individual working phase already, students in the systematic arm started to ask questions, make comments and vividly discuss their (virtual) patients with the teacher. By contrast, the KF arm students, in the individual working phase, appeared to be completely absorbed by their task and virtually asked no questions at all. This suggests that the students in the KF arm focused more individually on their task in order to work through their cases in time, while the students in the systematic arm obviously had more freedom to explore their respective cases, used the opportunity to interact with each other and thus, reflected more deeply on the content. Additional support for this impression comes from the evaluation survey. The results indicate that the students in the KF arm experienced more time pressure and a higher work-load, and that they perceived the material as more difficult.

The bottom line is that while the students in the systematic arm obviously felt that they had enough time to work through the new content and method, the KF arm students faced a high cognitive load, some of them maybe even an overload, such that it might have slightly compromised their learning outcomes. This raises the question whether our implementation of the key features principle could be optimized: Maybe the additional cognitive task of extracting the key features in a very short time, even though we tried to deliberately highlight them through the instruction, was too demanding for some KF arm students.

Another possible explanation for our results is that the group discussion – which was identical in both arms – has levelled out potential differences. However, an alternative study design would have led to other problems: Had the group discussion been different in the two arms, a possible confounding factor would have been introduced, especially given the fact that the teacher was not blinded. Had there been no group discussion at all, then the whole endeavor of using VPs for teaching would have become unstructured and questionable: In order to extract a relevant learning experience and to gain a deeper understanding, it is essential for students to reflect on the cases they have seen, to receive feedback on their thoughts and to discuss them with others [4, 23, 43]. Hence, we regard this design issue as a strength of our study since this makes it more realistic.

Yet another possible interpretation of our results is that the systematic way of approaching cases has indeed advantages for novice learners. The above-mentioned observations from the evaluation survey suggest that the learning style implemented in the systematic arm, in giving the students more freedom for “experimentation” and for deeper reflection, leads to a kind of knowledge that makes them feel safer and more comfortable with the material. Since they scored equally well on our SCT although they had seen fewer cases, it might also suggest that their knowledge is more flexible and more readily transferable to other clinical problems. Hence, it might be true that learners simply need enough time to explore new terrain themselves, and that there is no easy short-cut to avoid this.

This would also correspond to the observation from clinical routine that in order to manage a certain number of patients on the ward, young and unexperienced residents usually need more time than experienced doctors. On the other hand, this might also reflect a lack of guidance and feedback for young residents. In fact, this is a well-known problem in high-workload clinical environments [4].

Taken together, our attempt to deliberately make students familiar with the key features of VP cases did not work the way we expected. Whether this is due to our specific implementation of the KF principle, or due to KF-based instruction itself, is not clear.

### Future research

We suggest to explore other implementations of the KF principle for instruction. Specifically, instead of the “post-hoc reduction” of originally detailed VP designs which we used in our KF trial arm, it could also be interesting to use VPs that are key feature-based by design, and to compare it to detailed ones. The corresponding idea would be not to use the same VP design and vary only the instruction – as we did –, but to vary the VP design itself.

Generally, more research is needed on how to implement VP-based learning in undergraduate medical education, given its high potential for the development of clinical reasoning skills.

## Conclusion

In conclusion, we have not been able to show empirically that working on multiple short and focused cases – the key feature principle – is more effective for the learning of clinical reasoning skills than working on few long and systematic cases in the curricular use of virtual patients. In this study powered for a medium effect, we could not observe a significant advantage of either teaching methodology. Possible explanations include the short duration of our study and the high cognitive load experienced by our KF arm students. Due to limitations of our investigation, the results should be confirmed by other studies.

More research is needed to investigate whether alternative implementations of key feature-based instruction are beneficial for the acquisition of clinical reasoning skills, and whether we can achieve long-term effects.

## Additional files

Additional file 1: Raw dataset (XLSX 26 KB).

Additional file 2: Explanation of terms and figures in the raw dataset (PDF 15 KB).

## Abbreviations

AGG: Aggregate (SCT scoring method)
CI: Confidence interval
CT: Computed tomography
ITT: Intention-to-treat (analysis)
KF: Key feature(s)
MC (Q): Multiple-Choice (Questions)
PP: Per-Protocol (analysis)
SCT: Script concordance test
SD: Standard deviation
VP: Virtual patient
