# Supplementary material for: Virtual patients in the acquisition of clinical reasoning skills: does presentation mode matter? A quasi-randomized controlled trial
Source: BMC Med Educ. 2017 Sep 15;17:165. doi: 10.1186/s12909-017-1004-2 (PMC5603058; doi:10.1186/s12909-017-1004-2)
Supplement: Supplementary file 2 — Explanation of terms and figures in the raw dataset (PDF 15 KB). [file 12909_2017_1004_MOESM2_ESM.pdf]

Explanation of terms and figures in the raw dataset

Category	Value	Meaning
„_varname“	item[xx_y]	SCT case no. [xx], question no. [y]
	eva[xx_yy]	Evaluation questionnaire: Subscale no. [xx], item no. [yy]
Participant no.	stud_short_[xx]	KF arm student no. [xx]
	stud_long_[yy]	Systematic arm student no. [yy]
„topic“	1	SCT subscale „Acute abdomen“
	2	SCT subscale „Gastrointestinal hemorrhage“
	+	Retain orientation of Likert scale (Evaluation questionnaire)
	-	Invert orientation of Likert scale on analysis (Evaluation questionnaire)
„group“	2	KF arm
	3	Systematic arm
„mc“		Score in multiple-choice test on visceral surgery (max. 40)
„treatment“	0	Did not receive treatment as intended, i.e. included in intention-to-treat, but not in per-protocol analysis
	2	KF arm; received treatment as intended, i.e. included in intention-to-treat as well as per-protocol analysis
	3	Systematic arm; received treatment as intended, i.e. included in intention-to-treat as well as per-protocol analysis
„ggroup“		Learning group no. as specified in the methods section of the manuscript
“additional_work” (Additional training after the seminar)	0	Unknown
	1	No
	2	Yes
